# Sexual history taking by doctors in primary care in North West province, South Africa: Patients at risk of sexual dysfunction overlooked

**DOI:** 10.4102/phcfm.v14i1.3238

**Published:** 2022-04-23

**Authors:** Deidré Pretorius, Ian D. Couper, Motlatso G. Mlambo

**Affiliations:** 1 Division of Family Medicine, School of Clinical Medicine, University of the Witwatersrand, Johannesburg, South Africa; 2 Ukwanda Centre for Rural Health, Faculty of Medicine and Health Sciences, Stellenbosch University, Stellenbosch, South Africa; 3 Department of Institutional Research and Business Intelligence, University of South Africa, Pretoria, South Africa

**Keywords:** sexual history taking, routine consultation, holistic practice, communication, patient-doctor engagement, diabetes, hypertension, sexual dysfunction

## Abstract

**Background:**

Sexual history taking seldom occurs during a chronic care consultation and this research focussed on consultation interaction factors contributing to failure of screening for sexual dysfunction.

**Aim:**

This study aimed to quantify the most important barriers a patient and doctor experienced in discussing sexual challenges during the consultation and to assess the nature of communication and holistic practice of doctors in these consultations.

**Setting:**

The study was done in 10 primary care clinics in North West province which is a mix of rural and urban areas.

**Methods:**

One-hundred and fifty-five consultation recordings were qualitatively analysed in this grounded theory research. Doctors and patients completed self-administered questionnaires. A structured workplace-based assessment tool was used to assess the communication skills and holistic practice doctors. Template analysis and descriptive statistics were used for analysis. The quantitative component of the study was to strengthen the study by triangulating the data.

**Results:**

Twenty-one doctors participated in video-recorded routine consultations with 151 adult patients living with hypertension and diabetes, who were at risk of sexual dysfunction. No history taking for sexual dysfunction occurred. Consultations were characterised by poor communication skills and the lack of holistic practice. Patients identified rude doctors, shyness and lack of privacy as barriers to sexual history taking, whilst doctors thought that they had more important things to do with their limited consultation time.

**Conclusion:**

Consultations were doctor-centred and sexual dysfunction in patients was entirely overlooked, which could have a negative effect on biopsychosocial well-being and potentially led to poor patient care.

## Introduction

Globally, doctors’ practices are directed by country-specific and consensus guidelines for sexual health.^1,2^ In South Africa, doctors should be steered by national guidelines for sexually transmitted infections and HIV, as well as best practice standards that combine the guidelines of the International Society of Sexual Medicine, and the recommendations of the International Consultation of Sexual Medicine-5 (ICSM-5), amongst others.^3,4^ In terms of these guidelines, sexual history taking is considered an essential element of clinical practice. Kok^5^ suggested that failing to take a sexual history, or not delving into sensitive discussion topics during the consultation, can be an omission of beneficence, and could even be considered a sign of negligence. When sexual history is not explored, many diabetic and hypertensive patients continue living with complications of their disease and medication, with no management of sexual challenges.^6,7,8,9^ In order to manage sexual challenges, doctors must screen for reproductive health issues, sexual risk behaviour, sexual dysfunction and sexual trauma.^5^ Screening for sexual dysfunction is also clinically prudent, because of the potential for serious comorbidities associated with it. For example, evidence shows that it can be a biomarker for cardiovascular health.^5,10^

A principle of family medicine is that every contact with the patient is an opportunity for health education and prevention of disease, which creates the expectation that screening will be conducted.^11^ Research suggests that only 10% – 58% of doctors globally conduct routine sexual history taking.^12,13^ A study in Nigeria found that 38% (*n* = 187) of doctors claimed that they would take a sexual history, even if the patient did not raise the issue.^14^ Less than 70% of doctors in Ireland felt comfortable discussing sex with patients.^15^ Conversely, Kingsberg^16^ postulated that 71% of American patients feared that their sexual complaints would be dismissed during a consultation. Common reasons given by physicians for not taking a sexual history are time constraints, experiencing or causing embarrassment, underestimating the prevalence and impact of sexual dysfunction, and different expectations of who must initiate such a discussion.^5,17,18^ Between 14% and 20% of patients disclose sexual dysfunction during a consultation.^19,20,21^ When patients’ complaints were often dismissed or their needs were not met, they experienced a low quality of life.^22,23,24^

Failing to offer a comprehensive or informed service to patients can lead to harmful compensatory behaviours. Globally, culture still shapes perceptions and life patterns, and it precipitates the use of traditional methods and practices to enhance sexual functioning, some of which may be harmful to the patient.^25,26^ A systematic review of studies on intimate partner violence and sexual health concluded that sexual risk-taking, infidelity, sexually transmitted infections and sexual dysfunction were associated with domestic violence.^27^ The consequence of non-diagnosis or non-management of sexual dysfunction is unquestionably detrimental to the patient’s biopsychosocial well-being.

This research was motivated by the consequences of sexual dysfunction, as well as the fact that the primary researcher did not observe screening for sexual dysfunction when students at a tertiary training facility carried out observed consultations. The occurrence and nature of sexual history taking in rural primary care settings in South Africa are required to be investigated. The research questions were what were the frequency and nature of sexual history taking during routine consultations; what single barrier to history taking did patients and doctors identify and did the doctors have good communication skills and practice holistically?

This study observed, described and sought to understand sexual history taking during routine chronic illness consultations in primary care settings in Dr Kenneth Kaunda Health District, North West province, South Africa. The objectives of the broader study were to determine if history taking for sexual dysfunction took place, and if so, to analyse it. The aim of the study related to this article intended to identify if patients wanted to discuss sexual issues with doctors, as well as the most important barriers a patient and doctor experienced in discussing sexual challenges during the consultation. Lastly, the study planned to assess the nature of communication and holistic (biopsychosocial) practice of doctors in these consultations.

## Methods

### Study design

This was part of a grounded theory PhD study analysing video-recorded consultations between primary care doctors and adult patients (> 18 years of age), who were at risk of sexual dysfunction due to their diagnosis of hypertension and/or diabetes.^28^ The study consisted of different components, namely, (1) the observation and analysis of recorded consultations, (2) determining the proportion of the patient participants living with symptoms of sexual dysfunction and (3) interviewing both doctors and patients on their perceptions of sexual history taking. This article describes the quantitative aspects of patient and doctor characteristics, the nature of communication and holistic practice and patient and doctor preferences and single barrier to sexual history taking. Questionnaires covered information regarding patient and doctor characteristics, as well as questions on their views regarding the disclosure of sexual challenges. Some questions allowed the doctors and patients to indicate how often the statement applies to them, and one question asked them to identify the most important barrier to sexual history taking.

### Setting

The setting, Dr Kenneth Kaunda Health District, in the North West province, South Africa, is a mixed rural versus urban setting with the biggest area described as rural with mining and agricultural activities. Dr Kenneth Kaunda District covers an area of 14 767 km^2^, has a total population of 807 252 and renders government financed primary care services to a population of 707 479.^29^ Patients have free access to primary care services. Nineteen per cent of the population are unemployed and 14% of the households live with R400.00 (approximately €22.00 [euro] or $27.00 [United States dollars]) and less per month at the time of the research, which meant they did not have the resources to consult privately.^29^

### Sample size and sampling

The sampling approach employed in this study was a stepwise process addressing the primary care clinics first, then the doctors and finally the patients. Primary care in South Africa is nurse-driven, and many clinics operate without doctors. For inclusion in this study, a clinic needed to have a doctor visiting at least once a week. Eleven out of 26 clinics and nine community health centres were identified as meeting this criterion, of which managers of two clinics declined permission for their facilities to be included, leaving nine facilities in the sample. All 28 doctors working in the selected healthcare sites at the time were recruited to participate in the study. Twenty-seven doctors initially agreed to participate, but when the actual moment of video-recording arrived, six withdrew. Of those who declined, one doctor did not honour his appointment on two occasions, without any reason; one committed and then avoided the researcher and the other four did not feel comfortable doing an observed consultation.

Once the doctors had been recruited and had consented to participate, patients were recruited. Consecutive adult patients living with diabetes and hypertension were purposefully selected, based on their theoretical high risk of sexual dysfunction in this population. Determining the number of consultations to observe sexual history taking events was based on the midpoint of the disclosure rate of sexual dysfunction if asked, which varies between 14% and 20%,^19,20,21^ and that formed the basis for sample size calculation.^30,31,32^ The sample size calculation was completed using an expected rate for history taking of 0.17 and an accuracy level of 0.06:


$$n=p(1-p){\left[1.96/\text{a}\right]}^{2}$$ [Eqn 1]

where *p* = proportion expected disclosure rate or history taking (0.17), and a = accuracy as a proportion (0.06), leading to an expected sample size of 151 consultations.

Five hundred and forty patients who consulted 21 doctors in 10 primary care sites over 9 weeks, during 2018 were informed about the study whilst sitting in the health facility waiting room. The trained research assistant (a student at a tertiary institution) recruited 236 consecutive patients after they collected their files and of these, 151 gave written consent to participate. Fifty-six patients declined participation and their reasons for declining participation were not documented, as participation was voluntary. Another 29 patients were excluded because of incomplete consent forms, non-completion of all the questionnaires or leaving before completing the full process.

### Data collection

The research assistant was responsible for recruitment, obtaining consent and research administration, such as completion of tracking sheets, as well as facilitating the completion of the demographic and sexual dysfunction questionnaires. The primary researcher was responsible for management of the recordings, the completion of the biographic questionnaire (clinical information from the patient file) with the doctor, and the management of the patients’ prescriptions, so that they were not delayed at the pharmacy. Both the primary researcher and research assistant were involved in the information sessions and kept field notes. The research assistant obtained consent for both video-recording of the consultation and the completion of the questionnaire following the consultation. Doctors and patients were informed that the content of the consultation would be analysed, without reference to the focus on sexual history taking in order to not influence the consultation outcome. Patients who were too sick to participate and those who left directly after the consultation without completing the questionnaire were excluded from the study. Video-recording was conducted using a laptop-mounted camera, which focused on the patient and doctor, excluding examination areas. Recording was activated and deactivated on entry and exit of each patient. The researcher was not present during consultations. The researcher ensured that the patient did not experience any delays, such as losing his or her place in the queue or extended waiting periods for medication, because of the research participation. Following the recording, the patient consented to complete questionnaires on sexual dysfunction^33,34,35^ and a demographic questionnaire. At the end of the day after all the recorded consultations, the doctors completed the demographic questionnaires. The research assistant speaks five of the national languages and assisted in the recruiting and consenting phase.

### Data analysis

The recorded consultations were first analysed using open and focussed coding. To support the initial observations and analysis, template analysis followed using the workplace-based assessment (WPBA)^36^ competence framework of the Royal College of General Practitioners. The WPBA^36^ competence framework of the Royal College of General Practitioners was used for the consultation interactions. The tool describes good practice standards for communication and holistic practice that formed a well-defined *a priori* code for template analysis. This assessment was done to assess two out of the 13 competencies of the WPBA tool, namely, good communication skills (14 sub-descriptors) and holistic practice skills (10 sub-descriptors) of the doctors in a standardised manner.^37^ The assessment tool has four categories for each competency with primary descriptors for every category: insufficient evidence, need further development, competent and excellent. The tool describes good practice standards for communication and holistic practice, which formed a well-defined *a priori* code for template analysis. Confidence intervals of 95% were calculated for all the percentages applicable to the WPBA outcomes. Descriptive statistics were used for patients’ and doctors’ characteristics, opinions on sexual challenges and disclosure thereof, frequencies of communication and holistic practice categories.

### Ethical considerations

The study was approved by the Human Research Ethics Committee (Medical) of the University of the Witwatersrand (M160557). Clinics were referred to only as site numbers to ensure confidentiality of both the setting and healthcare workers. The Directorate: Policy, Planning, Research, Monitoring and Evaluation of the Department of Health, North West province, South Africa granted permission for the research to proceed. Permission to use the sexual dysfunction questionnaires was granted by the publishers. Data were stored and password protected on an external hard drive at the university.

## Results

### Doctors’ and patients’ characteristics

Twenty-one (75%) of the recruited 28 doctors participated. As reflected in Table 1, the doctor participants consisted of 15 men (71%) between the ages of 25 and 67 years (median 39), and six women (29%) aged 25–34 years (median 28 years). Four doctors (19%) were African first language speakers and three (14%) shared the indigenous language to the area, namely Setswana. The male doctors had longer work experience than the female doctors (median of 144 months vs 36 months).

**TABLE 1:** Demographic data of patient participants.

Characteristics	Male patients† (*n* = 47)		Female patients‡ (*n* = 104)		Male doctors§ (*n* = 15)		Female doctors¶ (*n* = 6)	
	*n*	%	*n*	%	*n*	%	*n*	%
**Age**	-	-	-	-	-	-	-	-
**Marital status**								
Divorced	3	6	5	5	-	-	-	-
Estranged	-	-	1	1	-	-	-	-
Live together	5	11	6	5			-	-
Married	17	36	38	37	9	60	2	33
No partner	1	2	23	22	-	-	-	-
Separated	2	4	-	-	-	-	-	-
Single	14	30	28	27	6	40	4	67
Widowed	6	13	27	26	-	-	-	-
**Highest level of Education**					-	-		
No education	-	-	1	1	-	-	-	-
Primary School (Grade 0–7)	19	40	42	40	-	-	-	-
Secondary school (Grade 8–12)	25	53	52	50	-	-	-	-
After school (college, university)	3	6	9	9	15	100	6	100
Diploma after MBChB	-	-	-	-	6	40	1	17
Another degree besides MBChB	-	-	-	-	3	20	3	50
**Home language**								
Afrikaans	-	-	13	13	3	20	3	50
English	-	-	1	1	-	-	2	33
French	-	-	-	-	6	40	-	-
IsiXhosa	11	23	9	9	-	-	-	-
IsiZulu	1	2	2	2	-	-	-	-
Mandarin	-	-	-	-	1	7	-	-
Sesotho	6	13	18	17	1	7	-	-
Setswana	29	62	61	59	3	20	1	17
Spanish	-	-	-	-	1	7	-	-

† , Range = 26–74 years; Mean = 45 years; Median = 49 years.

‡ , Range = 19-93 years; Mean = 53 years; Median = 55 years.

§ , Range = 25–67 years; Mean = 42 years; Median = 39 years.

¶ , Range = 26–34 years; Mean = 29 years; Median = 28 years.

Forty-seven male patients (31%) and 104 female patients (69%) of the 540 recruited patients (64% response rate) participated in this study (Table 1). The patients had a normal age distribution, and the sample passed the Shapiro-Wilk test for normality. Only 8% of the participants completed secondary school and/or had tertiary qualifications; 41% had no schooling or attended only primary school. Most of the participants were Setswana speaking, as expected due to the population distribution in the district. The patients were familiar with the clinics and clinic staff, and only 10 patients (7%) consulted at that clinic for the first time.

### Sexual history taking and the consultation

Sexual dysfunction was not elicited or diagnosed during the consultations, despite patients living with sexual dysfunction symptoms. Doctors did not ask any questions that could lead to the discovery or exploration of sexual dysfunction. One female patient presented with pain after intercourse, however, the doctor still did not consider sexual dysfunction as a differential diagnosis. The patients also did not raise sexual challenges.

### Communication and consultation skills

On an average, communication and consultation skills were assessed as needing further development. Communication with patients was limited to developing a functional working relationship, with the presenting complaint rather than the patient being the focus of the consultation (Table 1). In 32 consultations (21%), the doctors’ performance could not be assessed due to insufficient evidence of communication during the consultation. In these consultations, patients were not greeted, and interactions were limited to instructions for examination, to collect medicine, to go for X-rays or to make a follow-up appointment. The observed trend in communication was that it was doctor-dependent, as the consultations conducted by a specific doctor followed the same communication pattern, irrespective of the patient in front of them.

The competency of practising holistically, that is, incorporating physical, psychological, social, socio-economic and socio-cultural elements, as well as taking feelings, thoughts and health promotion into account, was not observed in 100 consultations (66%) (Table 3). In 17 consultations (11%), doctors demonstrated competency in holistic practice and 34 (23%) needed further development.

**TABLE 2:** Consultation competency according to the workplace-based assessment framework for communication and consultation skills.

Competency level	Descriptor	Number of consultations (*n* = 151)		
		*n*	%	CI (%)
Insufficient evidence of communication and consultation skills	From the available evidence, the doctor’s performance cannot be placed on a higher point of this development scale.	32	21	15 – 28
Needs further development of communication and consultation skills	Develops a working relationship with the patient, but one in which the problem rather than the person is the focus.	54	36	29 – 44
	Produces management plans that are appropriate to the patient’s problem.	33	22	16 – 29
	Provides explanations that are relevant and understandable to the patient, using appropriate language.	14	9	6 – 15
	Achieves the tasks of the consultation but uses a rigid approach.	-	-	-
Competent communication and consultation skills	Explores the patient’s agenda, health beliefs and preferences. Elicits psychological and social information to place the patient’s problem in context.	18	12	8 – 18
	Works in partnership with the patient, negotiating a mutually acceptable plan that respects the patient’s agenda and preference for involvement.	-	-	-
	Explores the patient’s understanding of what has taken place.	-	-	-
	Flexibly and efficiently achieves consultation tasks, responding to the consultation preferences of the patient.	-	-	-
Excellent communication and consultation skills	Incorporates the patient’s perspective and context when negotiating the management plan.	-	-	-
	Whenever possible, adopts plans that respect the patient’s autonomy.	-	-	-
	Uses a variety of communication techniques and materials to adapt explanations to the needs of the patient.	-	-	-
	Appropriately uses advanced consultation skills such as confrontation or catharsis to achieve better patient outcomes.	-	-	-

*Source:* From WPBA Capability Framework. Royal College of General Practitioners [homepage on the Internet]. No date. [cited 2021 Feb 18]. Available from: https://www.rcgp.org.uk/training-exams/training/mrcgp-workplace-based-assessment-wpba/wpba-capability-framework.aspx

**TABLE 3:** Consultation competency according to the workplace-based assessment framework for practising holistically.

Competency level	Descriptor	Number of consultations (*n* = 151)		
		*n*	%	CI (%)
Insufficient evidence practising holistically, promoting health and safeguarding	From the available evidence, the doctor’s performance cannot be placed on a higher point of this development scale.	100	66	58 – 73
Needs further development practising holistically, promoting health and safeguarding	Enquiries into both physical and psychological aspects of the patient’s problem.	9	6	3 – 11
	Recognises the impact of the problem on the patient.	14	9	6 – 15
	Uses him or herself as the sole means of supporting the patient.	11	7	4 – 12
Competency of practising holistically, promoting health and safeguarding	Demonstrates understanding of the patient in relation to his or her socio-economic and cultural background.	10	7	4 – 12
	Additionally, recognises the impact of the problem on the patient’s family/carers.	7	5	2 – 9
	Utilises appropriate support agencies (including primary healthcare team members) targeted to the needs of the patient.	-	-	-
Excellence of practising holistically, promoting health and safeguarding	Uses this understanding to inform discussion and to generate practical suggestions for patient management.	-	-	-
	Recognises and shows understanding of the limits of the doctor’s ability to intervene in the holistic care of the patient.	-	-	-
	Organises appropriate support for the patient’s family and carers.	-	-	-

*Source:* WPBA Capability Framework. Royal College of General Practitioners [homepage on the Internet]. [Date unknown] No date. [cited 2021 Feb 18]. Available from: https://www.rcgp.org.uk/training-exams/training/mrcgp-workplace-based-assessment-wpba/wpba-capability-framework.aspx

Despite important observations, there were no statistical associations between patient and doctor characteristics, practising holistically or communication skills and sexual history taking.

### Doctors’ and patients’ opinions on discussion of sexual dysfunction

Most of the patients (58%) were willing to discuss sexual challenges with the doctor. Nine participating doctors (43%) said that they would like to discuss sexual matters with patients. Both participant groups preferred that the other party initiates the discussion of sexual challenges, where 29% of the doctors expected the patient to initiate the discussion and 20% of patients expected the doctor to ask. No statistical association was found between demographic factors and whom participants believed must initiate the discussion.

Most of the patients (77%) did not identify a barrier to sexual history taking. They reported that nothing would stop them in their need to express sexual challenges to their doctors, whilst 67% of the doctors expressed that they either did not think about sexual dysfunction or had too many other things to cover in the limited consultation time (Table 4). Fifteen patients (10%) valued a receptive attitude and considered a friendly, approachable doctor who listens to be someone to whom they would likely disclose sexual challenges. Five (3%) patients considered that consulting a different doctor every time they came to the clinic to be a barrier to sharing sexual challenges.

**TABLE 4:** Doctors’ and patients’ opinions on discussion of sexual dysfunction.

Opinions	Patient participants (*n* = 151)		Doctor participants (*n* = 21)	
	*n*	%	*n*	%
**I would like to discuss sexual matters during the consultation**				
Yes, always	84	56	9	43
Yes, sometimes	3	2	-	-
Only if the doctor asks	30	20	4	19
Only if the patient asks	29	19	6	29
No never	5	3	1	4
**Perceived single most important barrier to discuss sexual challenges in the consultation**				
None, nothing will stop me	116	77	-	-
Doctor has a negative attitude (rude, unfriendly, does not listen)	12	8	-	-
Shyness/nervousness	6	4	-	-
Lack of privacy (nurse, translator or other person in the room)	5	3	-	-
Always a different doctor (lack of continuity of care)	4	3	-	-
If the doctor does not show interest and ask about my problems	3	2	-	-
Sex/gender of the doctor	3	2	-	-
S/he will not understand cultural stuff (e.g. going to the mountain)	2	1	-	-
Too many other more important things to ask	-	-	7	34
Time constraints	-	-	4	19
Do not think of sexual dysfunction	-	-	3	14
Lack of knowledge on subject	-	-	2	10
Gender or culture	-	-	1	5
Not presenting complaint	-	-	1	5
Medico-legal implication for bridging privacy	-	-	1	5
Against personal value system	-	-	1	5
Sexual dysfunction is not indicated	-	-	1	5

**Total**	**151**	**100**	**21**	**100**

Table 4 includes a few isolated but disturbing findings, such as that one doctor believed it to be illegal to ask about sexual dysfunction, and another considered it against his personal value system to ask about sexual challenges if the patient was not married.

## Discussion

This study set out to observe sexual history taking during routine chronic illness consultations in primary care settings. There was no history taking for sexual dysfunction in this current study. Yet, 94% of female patients were living with sexual challenges and 98% of male patients were living with erectile dysfunction.^38^ This leaves the question as to why time in such a routine consultation could not be used to explore comorbidities, such as sexual dysfunction, or psychosocial factors related to the illness. This type of patient–doctor interaction would perhaps be associated with holistic practice; however, assessment of the consultations reflected that most doctors did not interact holistically with their patients.

Research suggests that a biopsychosocial and culturally sensitive approach, that is characteristic of a holistic practice, can contribute to sexual history taking.^39,40,41^ Good communication is required to facilitate holistic practice, whereas doctors displayed reasonable communication skills in only 12% of the consultations. One understands that sexual functioning is a sensitive matter, and that it might elicit discomfort if the doctor does not know how to start the conversation.^42^ Andrews has suggested using a sensitive non-judgemental approach with open-ended questions to approach this discussion, but such an approach assumes that doctors can work on reasonable communication skills in order to adopt it.^43^ In this study, extremely poor communication was observed, which would explain the poor level of interaction between patients and doctors in general, and, especially, the absence of communication around sexual dysfunction. If communication is poor, it clearly leads to missed opportunities for exploring sexual well-being.^44^

Communication around sexual health was also limited by common barriers to sexual history taking. Participants chose a single important factor that prohibited them from discussing sexual challenges with the doctor. Although low on their lists, sex, age and culture differences were identified and are well described in other research.^18,45,46^ The cultural aspects identified by the patients in this study were related to traditional practices, often ancient, that enable a connection to ancestral spirits when patients are sick.^47^ Going to the mountain, mentioned by one patient, is an example of such a practice. Although African patients often consult traditional healers for herbal medicine to counter sexual dysfunction or to improve pleasurable sex, these aspects were not part of the scope of this study.^26,48,49^ It is, however, important to note that cultural barriers for patients are more than just cultural or normative prejudice that leads doctors to avoid addressing the sensitive topic of sexuality. In terms of the patient–doctor interaction, this links with the holistic practice of the doctors, or, in this case, the lack thereof.

It was noteworthy that most of the doctors in this study did not consider sexual challenges a priority, whilst patients expressed hesitation to share the information, as they did not perceive the doctors as open and friendly. A doctor-centredness in the relationship was observed, where doctors did not consider asking patients openly what they considered to be important, as well as what issues were troubling them, which might have elicited discussion on any other challenges. This approach is counterproductive in quality patient care, adherence to therapy, patient satisfaction and trust.^50^ Considering the three discourses of patient-centredness, namely ‘caring for patients’, ‘empowering patients’ and ‘being responsive’, these consultations failed to care for or empower patients.^51^ Doctors were not responsive to patient needs, which excluded the patient from decision-making and participating in self-care. Specifically, relevant to this study is that the lack of patient-centredness is detrimental to positive psychosocial outcomes.^50^ Even in a context of nursing, it was noted that the biomedical model hardly ever prioritises sexual concerns and these were often unacknowledged.^52^

A few patients also mentioned aspects such as time constraints, shame, embarrassment and lack of knowledge as barriers to discussing sexual challenges, which is also well described in the literature.^7,16,46,53,54^ Whilst patients verbally felt that nothing would stop them from talking to the doctor about their sexual challenges, they still lived with sexual dysfunction and did not raise it. One should consider that they may have raised it in the past, or that it did not occur to them to raise it with the doctor. Help-seeking behaviour plays a role in talking about sexual dysfunction. In an Iranian study, 36% of female patients did not think of seeking help.^55^ There is also the matter of low literacy levels and the understanding of illness and disease. A study in Ghana highlighted the fact that patients often do not know that their sexual challenges can be a medical condition.^56^ It may be that, although the patients were comfortable talking about it, they did not know that they could raise it with the doctor. Alternatively, acquiescence contributed to this failure to interact. Patients learned to fit in with their expected role as determined by doctor-centred care.^57^

The participants were purposively selected and deliberately biased towards the possibility of experiencing sexual dysfunction due to the diagnoses of hypertension and diabetes. Despite the risk for sexual dysfunction in these patients, and one patient presenting with a symptom associated with sexual dysfunction, none of the doctors screened for it. This is contrary to 43% of the doctors in this study who said that they were always ready to discuss sexual health issues during the consultation. One must then assume that there are other factors involved, and that doctors believe that they should be open to such discussions, even if they do not make them happen in practice. Such a mismatch between belief and action is not uncommon but is worth further exploration. The barriers doctors mentioned related to the fact that doctors did not consider screening to be indicated in these patients, or they did not think of sexual dysfunction. Research has suggested in the past that the lack of awareness of how illness influences sexual functioning can be a barrier for screening.^7,58^ Personal sexual agendas, professional attitude and an indifference to finding solutions are not often raised by doctors themselves, but a qualitative study in Malaysia suggested that it may impact negatively on sexual history taking.^46^ Regardless, the doctors overlooked the opportunity to screen for sexual dysfunction during the routine consultations in this study. The broader work of this research recognised that the patient, doctor and the health system contributed to the lack of patient-centredness.^59^ It also generated systemic questions such as the patient’s help-seeking behaviour and doctors’ training about sexual health matters that need to be explored in future.

External validity of quantitative data due to a small sample size is a limitation in this study. However, as the quantitative data were mainly in the interest of triangulation of a qualitative study, the strength of the study lies in the methodology of having various sampling points of different aspects and methods to describe the phenomenon of real-time sexual history taking in routine consultations with patients at risk of sexual dysfunction. The recording of the consultations could be perceived as a limitation in a way that one would expect best practice standards. However, research suggested that recording of consultations has a minimal influence on behaviour patterns.^60^

This research not only demonstrates the poor-quality care for sexual health that arises from doctor-centredness, but also demonstrates omissions in communication and holistic practice, which potentially have negative implications for general patient care. In all routine consultations, particularly in those involving patients living with chronic illnesses, the doctor is needed not only for providing a repeat prescription, but also for addressing any ongoing unspoken challenges that should surface. Given the gap between theory and practice evidenced in the study, this will require an attitude change on the part of the doctors involved. Guidelines that address history taking for sexual dysfunction can facilitate awareness and attitude changes. Such a change will involve doctors dealing with their self-centredness to build relationships with their patients and nurture patient participation. It requires an awareness of and respect for their patients and associated holistic well-being. Most of all, it requires ongoing training in communication, so that doctors can initiate and maintain these sensitive discussions. If doctors are in doubt, all they need to do is ask their patients – the patients have made it clear that they will tell them.

## Conclusion

Consultations were doctor-centred and sexual dysfunction in patients was entirely overlooked, which could have a negative effect on biopsychosocial well-being and potentially led to poor patient care.
